# Estimation of SIR model’s parameters of COVID-19 in Algeria

**DOI:** 10.1186/s42269-020-00434-5

**Published:** 2020-10-19

**Authors:** Mohamed Lounis, Dilip Kumar Bagal

**Affiliations:** 1 Department of Agro-Veterinary Science, Faculty of Natural and Life Sciences, University of Ziane Achour, BP 3117, Road of Moudjbara, Djelfa, 17000 Algeria; 2 Department of Mechanical Engineering, Government College of Engineering, Kalahandi, Bhawanipatna, Odisha 766002 India

**Keywords:** COVID-19, Algeria, Python, SIR model, Reproduction number

## Abstract

**Background:**

The coronavirus disease 2019 (COVID-19) is reported in Algeria on February 25th, 2020. Since then, the number is still increasing leading to a total number of 36,699 cases and 1333 deaths on August 12th, 2020. Thus, comprehension of the epidemic curve is very important to predict its evolution and subsequently adapt the best prevention strategies. In this way, the current study was conducted to estimate the parameters of the classical SIR model and to predict the peak of the COVID-19 epidemic in Algeria using data from February 25th, 2020 to August 12th, 2020.

**Results:**

Results showed that the peak of the epidemic will be reached on September 8th, 2021 and the total infected persons will exceed 800,000 cases at the end of the epidemic. Also, more than 15 million persons will be susceptible. The reproduction number (*R*_0_) is estimated at 1.23254.

**Conclusion:**

These results may be helpful for the Algerian authorities to adapt their strategies and may be taken into consideration in the future phase of discontainment.

## Background

**Fig. 1:**
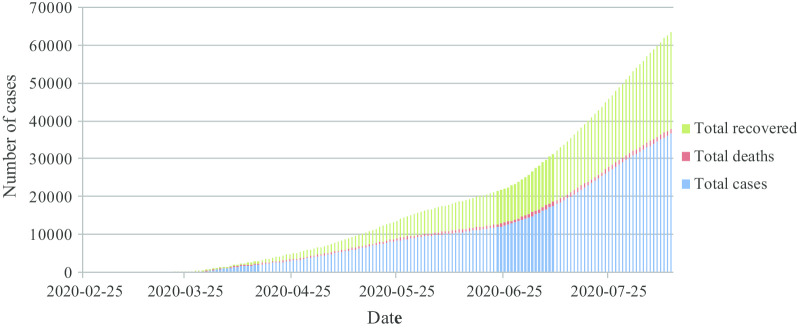
Total positive cases, deaths and recovered cases in Algeria

The coronavirus disease (COVID-19) is a respiratory viral disease first reported in Wuhan City, in China on December 2019 and turned epidemic worldwide (Lounis 2020). This disease has affected more than 21 million persons through the world leading to more than 6.000 deaths (JHUM 2020). COVID-19 has been declared in Algeria on February 25th, 2020 in the South department of Ouargla, and it continues to spread in the country afflicting a total number of 36,699 cases on August 12th, 2020. The total positive cases, deaths and recovered persons in Algeria are shown in Fig. 1 (Algerian Heath Ministry 2020).

**Fig. 2:**
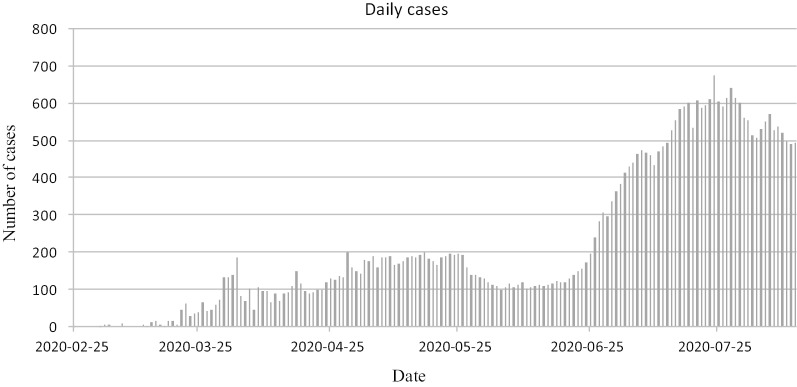
COVID-19 cases of Algeria as per time series from 25th February 2020

Regarding daily reported cases as shown in Fig. 2, we remark that the number of cases seemed to decrease after a peak in late April to middle May. This may be due to the measures implemented since March 12th, 2020. These interventions consisted of border banning, school closure, social distancing and cancellation of all public gatherings (Lounis 2020).

However, since middle June a considerable increase in daily reported cases was observed. Increased numbers have been reported due to different factors including the increase in daily performed tests but also the remarkable relaxation and lighting in prevention measures associated with the alleviation of the containment measures. This number is still increasing despite the strengthening of these measures in some departments. In this way, the prediction of COVID-19 evolution is of great importance for providing information for the public authorities that could help them to adopt or readapt their prevention measures. Multiple forecasting methods were used to analyze and predict the future trends of COVID-19 such as the logistic growth model (Zou et al. 2020), stochastic susceptible–infectious–removed (SIR) model (Bagal et al. 2020), susceptible–exposed–infectious–removed (SEIR) model (Al-Sayed et al. 2020) and natural growth model (Huang and Qiao 2020).

In this work, the classical SIR model has been utilized to predict the number of case counts, the rate of transmission, the reproduction number (*R*_*0*_), the size and the extinction date of the COVID-19 epidemic in Algeria.

## Methods

### Estimation of SIR model’s parameters

**Fig. 3:**
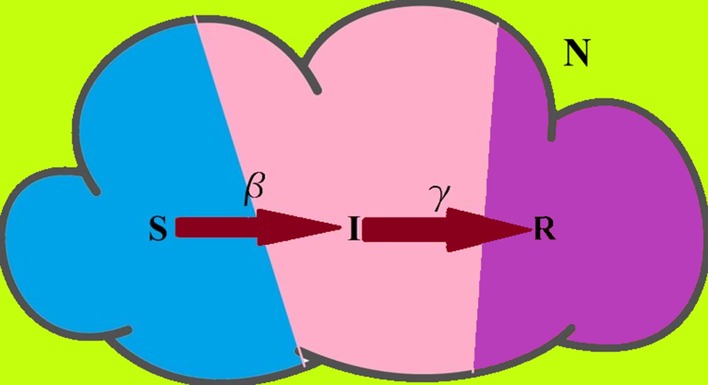
Scheme of the fraction of the SIR model

In the current study, the standard susceptible–infectious–recovered (SIR) model was adopted. In this model, we divide the total population (N) into three categories: *Susceptible (S), Infected (I) and Recovered (R).* The susceptible is the part of the total population which is vulnerable and is at risk of being infected (if previously unexposed to the pandemic disease); the infected is the fraction that has been infected (currently colonized by the pandemic disease), and the recovered is the fraction of the total population having recovered/removed (either by death or recovery). The basic structure of SIR mathematical model is described in Fig. 3 (Bagal et al. 2020).

The specific description of the SIR model is as follows:

1 $$\frac{{{\text{d}}S}}{{{\text{d}}t}} = - \frac{\beta }{N}{\text{SI }}$$

2 $$\frac{{{\text{d}}I}}{{{\text{d}}t}} = \frac{\beta }{N}SI - \gamma I$$

3 $$\frac{{{\text{d}}R}}{{{\text{d}}t}} = \gamma I$$

where *β* represents the effective transmission rate and *γ* represents the removal or the recovery rate. *γ* is defined as the inverse of the duration of recovery *d* (*γ* = 1/*d*). Knowing that the total population size (N = S + I + R) is time independent. The population of Algeria is estimated at 43,851,044 inhabitants in the middle of the year according to United Nations reports (Worldmeter 2020) which is the (N) value for the SIR modeling.

Initially, in the absence of infection we have I + R = 0 and S≈N. We obtain the following equation from Eq. (2):

4 $$\frac{{{\text{d}}I}}{{{\text{d}}t}}\sim I\left( {\beta - \gamma } \right)$$

Then, integration of Eq. (4) gives the Equation:

5 $$I = I_{0} e^{{\left( {\beta - \gamma } \right)t}}$$

### Determination of transmission rate (*β*) and the *m* (*m* = *β*−*γ*) value

When the infection occurs, the susceptible population is almost equal to the total population (S ≈ N). Subsequently, the number of infectious individuals *I*(*t*) first increases exponentially.

6 $$\frac{{{\text{d}}I}}{{{\text{d}}t}}\sim mI$$

*m* is the difference between transmission and recovery rates (*m* = *β*−*γ*.

7 $$I\left( t \right) = I_{0} e^{mt}$$

8 $${\text{In }}I = mt + {\text{In}} I_{0}$$

We can calculate the value of m from the log-plot data and for the best-line fit we can use, for example, the least squares.

In the current work, the used data file was considered from the JHU-CSSE (Johns Hopkins University Center for Systems Science and Engineering) 2019 Novel Coronavirus Visual Dashboard database (JHU-CSSE 2020). The simulation of the SIR model’s Python code (SMPC 2020) based on the dataset of Algeria was done on the Google Colab platform, and the online date calculator was used to estimate the futuristic dates (DC 2020).

### Determination of the recovery rate (*γ*)

If we suppose that *I* is a constant (*I*(*t*) =* I*_0_), we obtain the equation:

9 $$\frac{{{\text{d}}R}}{{{\text{d}}t}} = \gamma I_{0}$$

By integrating Eq. (9), then it is found the following Equation:

10 $$R\left( t \right) = \gamma tI_{0}$$

If time of recovery*t* = T days, and $R\left(T\right)={I}_{0}$*,* or $\gamma T=1$.

Therefore, we obtain the next Equation:

11 $$\gamma \approx \frac{1}{T}$$

when time change d*t* = *α*, we obtain the following Equation from Eq. (3):

12 $$\frac{{R\left( {t + \alpha } \right)}}{\alpha } = \gamma I$$

or

13 $$\gamma \approx \frac{{R \left( {t + 1} \right) - R\left( t \right)}}{I\left( t \right)}$$

Estimation of *I*_max_(percentage infected persons at the peak of the epidemic) and *S*_inf_ (percentage of susceptible people remaining after the end of the epidemic):

when we divide Eq. (2) by Eq. (1), we get the equation that follows:

14 $${\raise0.7ex\hbox{${\left( {\frac{{{\text{d}}I}}{{{\text{d}}t}}} \right)}$} \!\mathord{\left/ {\vphantom {{\left( {\frac{{{\text{d}}I}}{{{\text{d}}t}}} \right)} {\left( {\frac{{{\text{d}}S}}{{{\text{d}}t}}} \right)}}}\right.\kern-\nulldelimiterspace} \!\lower0.7ex\hbox{${\left( {\frac{{{\text{d}}S}}{{{\text{d}}t}}} \right)}$}} = - 1\frac{\gamma }{\beta } N \frac{1}{S}$$

or

15 $$\frac{{{\text{d}}I}}{{{\text{d}}S}} = - 1 + \frac{\gamma }{\beta } N\frac{1}{S}$$

If we integrate both sides, we obtain the Equation:

16 $$I = - S + \frac{\gamma }{\beta } N\log S + C$$

*C* is a constant.

Knowing that at the beginning of the infection, S≈N and the number of infected (I) is extremely low, this mean that At: $t=0, I\sim 0$ and $S\sim N$

Therefore, if we substitute these values in Eq. (16), we get:

17 $$0 = - N + \frac{\gamma }{\beta }N\;In\;N + C$$

or

18 $$C = {\text{N}}\left( {1 + \frac{{\upgamma }}{{\upbeta }}{\text{InN}}} \right)$$

From Eq. (18), we take the value of *C* and we change it into Eq. (16). We will obtain Eq. (19):

19 $$I = N - S + \frac{\gamma }{\beta } N {\text{In}}\frac{S}{N}$$

Equation (19) is valid for all times. In general, the number of infected persons (*I*) increases exponentially from the beginning of the infection to reach a peak, before gradually shrinking to zero. We will estimate the percentage infected persons at the peak of COVID-19 (*I*_max_) and remained susceptible people after the end of the epidemic.

Here, we use Eq. (19) to resolve Eq. (2) related to infection rate.

For simplification, we suppose $\mathrm{S}=\mathrm{Ns},\mathrm{ I}=\mathrm{Ni},$ $\mathrm{S}=\mathrm{Ns},\mathrm{I}=\mathrm{Ni},$ and $\mathrm{R}=Nr$ $\mathrm{S}=\mathrm{Ns}$ $\mathrm{S}=\mathrm{Ns}$.

where* s* is the fraction of total susceptible population.

*i* is the fraction of total infected population.

*r* is the fraction of total recovered/removed population.

Therefore, following equations are obtained:

20 $$\frac{{{\text{d}}t}}{{{\text{d}}t}} = \beta is - \gamma i = i\left( {\beta s - \gamma } \right)$$

and

21 $$i = 1 - s + \frac{\gamma }{\beta }{\text{In}} S$$

At the peak of the infection when d $\mathrm{di}/\mathrm{dt}=0$ $\mathrm{di}/\mathrm{dt}=0$ $\mathrm{di}/\mathrm{dt}=0$ $\mathrm{di}/\mathrm{dt}=0$, *s* is calculated by the equation that follows:

22 $$s = \frac{\gamma }{\beta }$$

if we substitute the value of s from Eq. (22) into Eq. (21), we get Eq. (23):

23 $$i_{{\max}} = 1 + \frac{\gamma }{\beta }\left( {{\text{In}}\frac{\gamma }{\beta } - 1} \right)$$

We, now, need to find out ${S}_{inf}=\underset{t\to \infty }{\mathrm{lim}}S(t)$ the percentage of remaining susceptible individuals after the end of the infection. It is noted that at the end of infection t tends to infinity (∞), and thus, i = 0. We can rewrite Eq. (21) as follows:

24 $$1 - s + \frac{\gamma }{\beta }{\text{In}} s = 0$$

We can solve Eq. (24) numerically to obtain the value of s.

### Determination of the basic reproduction number (R_0_)

*R*_0_ or the basic reproduction number is defined as the average number of people infected by a single individual. Mathematically, it represents the ratio of transmission and recovery rates.

25 $$R_{0} = \frac{\beta }{\gamma }$$

To calculate *R*_0_, we used the following Equations:

26 $$i_{{\max}} = 1 - \frac{1}{{R_{0} }}\left( {1 - {\text{In}}\frac{1}{{R_{0} }}} \right)$$

and

27 $$R_{0} = 1 - \frac{{{\text{In}}S_{{\inf}} }}{{S_{{\inf}} - 1}}$$

## Results

In this work, we applied a SIR model to estimate different parameters related to COVID-19 epidemic curve for Algeria.

**Fig. 4:**
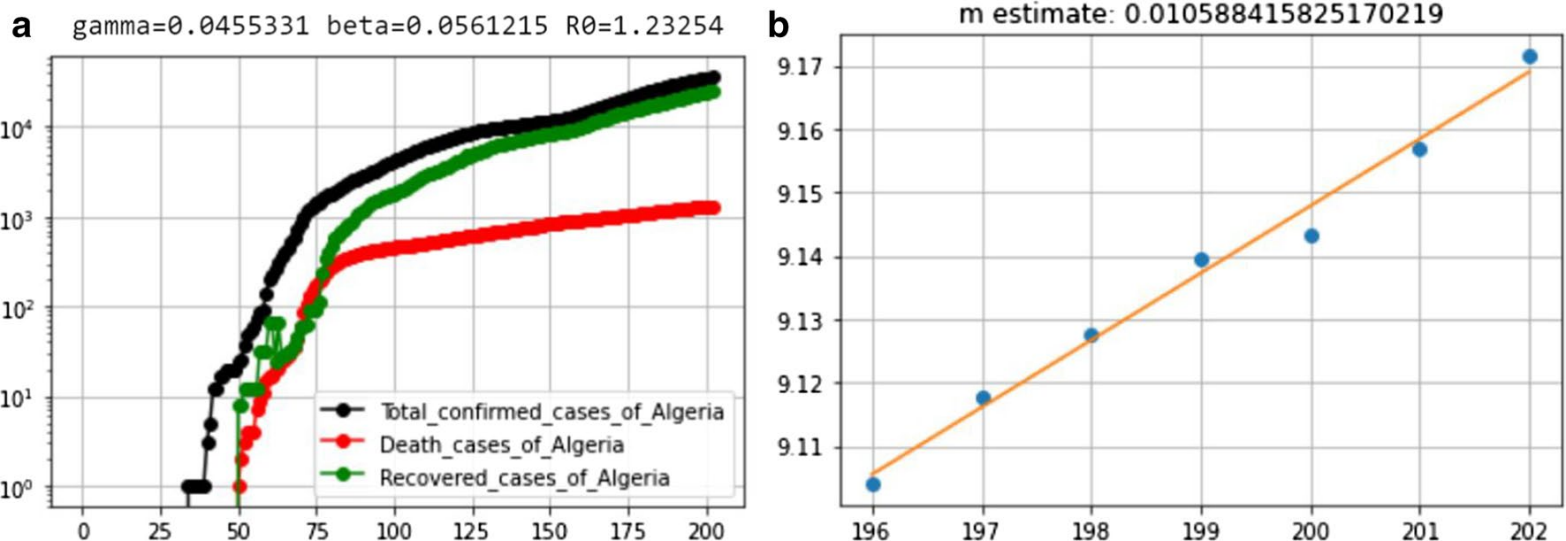
**a **Total confirmed cases, deaths and recovered cases in Algeria with “Gamma,” “Beta” and “R_0_” parameters of SIR model. **b** Plot for “m” parameter of SIR model in case of Algeria

The values of the rate of transmission (*β*) and the constant m obtained are shown in Fig. 4.

**Fig. 5:**
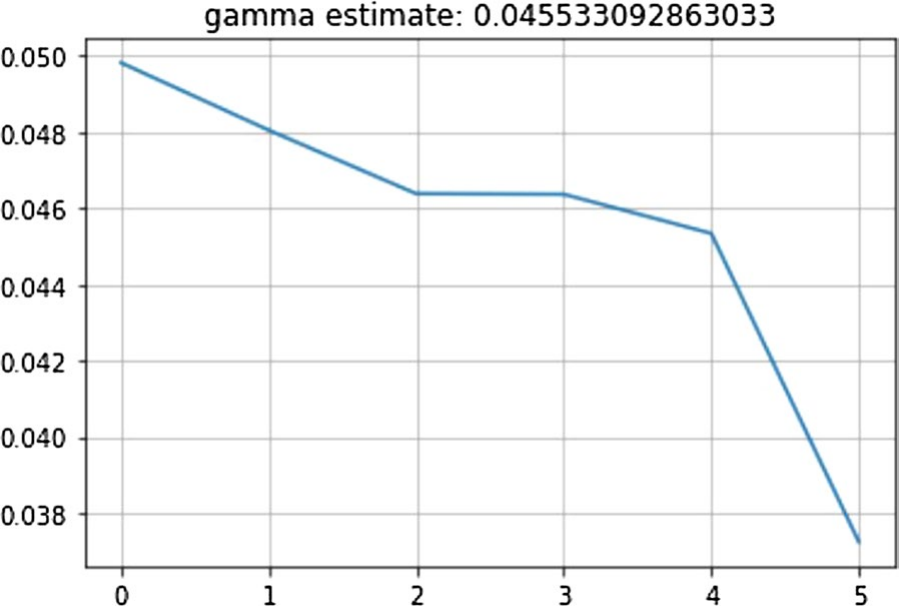
Plot for “γ” parameter of SIR model in case of Algeria

By approximating directly from the dataset of Algeria, we obtained the value of gamma plotted in Fig. 5.

The value of *I*_max_ was estimated at 1.91%. Taking into account the total number of the Algerian population, the number of sick peoples will be 8,37,555 persons (1.91% of 43,851,044).

**Fig. 6:**
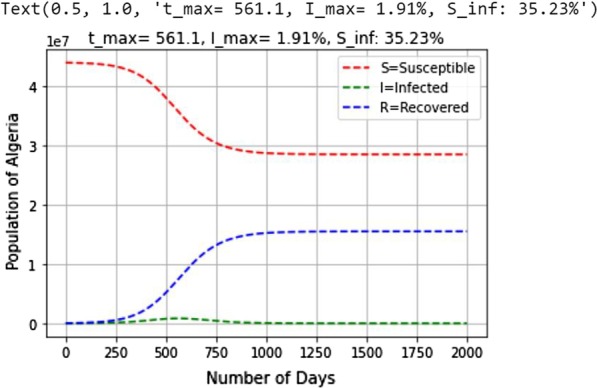
Estimated values of *t*_max_, *I*_max_ and *S*_inf_ in Algeria

The values of *S*_inf_obtained for Algeria are 35.23% (Fig. 6).

Thus, the number of susceptible people remaining after the end of the COVID-19 epidemic in Algeria will be 15,448,723 persons (35.23% of 43,851,044).

According to Fig. 6, we can also see that the peak of the epidemic *t*_max_ will be reached after approximately 561 days, i.e., 8th September 2021 (predicted date) from its emergence (February 25th, 2020) in Algeria.

At last, from the dataset of Algeria, we obtained the values of *R*_0_ = 1.23 which is shown in Fig. 4.

## Discussion

We estimated the different SIR parameters for COVID-19 in Algeria using data reported by the Algeria health ministry and the Johns Hopkins Institute dataset. This model which allows a prediction of a long period showed that the peak of the epidemic will be reached on September 2021. The basic reproduction number is estimated at 1.23 which is lower than that reported in India using the same model by Bagal et al. 2020.

At last, these parameters are estimated using only RT-PCR confirmed cases knowing that the number of tests do not exceed 2500 tests / day and the hospitalized patients diagnosed with CT scan is higher which could be considered as a limitation of the epidemic predictions.

## Conclusion

In this study, we estimated the parameters of SIR models for the COVID-19 epidemic in Algeria basing on the daily reported/confirmed cases by the Algerian Ministry of Heath from February 25th, 2020 to August 12th, 2020.The SIR model showed that the peak of the epidemic will be reached after an estimated period of 561 days from the beginning of the epidemic. The model estimated that 1.91% of the total population will be affected and the percentage of susceptible people remaining after the end of the infection will be about 35.23%

## Data Availability

The datasets used and/or analyzed during the current study are available from the corresponding author on reasonable request.

## Abbreviations

SIR model: Suspected–infected–recovered model
COVID-19: coronavirus disease 2019
